# Effect of deficit irrigation combined with *Bacillus simplex* on water use efficiency and growth parameters of maize during vegetative stage

**DOI:** 10.1186/s12870-024-04772-8

**Published:** 2024-02-26

**Authors:** Haq Nawaz, İlknur Akgün, Ulaş Şenyiğit

**Affiliations:** 1 Department of Field Crops, Faculty of Agriculture, Isparta University of Applied Sciences, Isparta, 32000 Turkey; 2 Department of Agriculture Structure and Irrigations, Faculty of Agriculture, Isparta University of Applied Sciences, Isparta, 32000 Turkey

**Keywords:** Deficit irrigation, *Bacillus spp.*, Water use efficiency, Growth attributes, Maize crop

## Abstract

The production of crops depending on many factors including water, nutrient, soil types, climate and crops types, water stress and drought is in one of the important factors affecting crop productivity. The experiment was conducted in pots to evaluate the effect of biofertilizers (*Bacillus simplex*) with deficit irrigations on the early development and growth of maize crop under greenhouse condition. Pre sowing seed was inoculated with strain of bacteria (B+/B-) and different irrigation levels (no stress: 100% (I_1_) and deficit irrigation: 75 (I_2_), 50 (I_3_), 25 (I_4_) % of required water amount to reach pot capacity) was performed. Data was collected on different morphological characteristics and root characteristic of maize crop. Highest plant height (125 cm), stem diameter (18.02 mm), leaf area (350 cm^− 2^), plant weight (180.42 g in fresh, 73.58 g in dry), root length (92.83 cm) root ((91.70 g in fresh, (28.66 g in dry) weight were recorded in pots applied with 100% irrigation followed by 75%. *Bacillus* treated plants showed significant increase in leaf area (214.20 cm^− 2^), plant fresh weight (91.65 g) and dry weight (42.05 g), root length (79.20 cm), root fresh (53.52 g) and dry weight (16.70 g) compared with control (without bacteria). Likewise highest relative water content of leaf was observed with I_3_ followed by I_2_ and I_1_ respectively. Highest water use efficiency was recorded as 0.67 g pot^− 1^ mm^− 1^ in I_1_ with B + treatment. Likewise, *Bacillus* inoculated pots resulted in increased water use efficiency (0.44 g pot^− 1^ mm^− 1^) compared with no application (0.36 g pot^− 1^ mm^− 1^). It can be endorsed from the outcome that *Bacillus* inoculation increased plant biomass, root biomass of maize and water use efficiency during early growth stage of maize despite of water stress and can be used under limited water condition for crop combating during moderate to lower stress conditions.

## Introduction

Maize is one of the vital cereals crops after wheat and rice grown in wide range of climates including tropical, subtropical and temperate regions of the world. Likewise other cereals crops maize crop also faces many biotic and abiotic constraints during life cycle. These include insect pest infestation, diseases, drought and nutrients deficiency [1]. Among these factors drought stress has considerable effect on crop growth and development [2]. Water is an important integral part of the plants, plays important role in maintaining growth, turgidity and acts as a reagent in plant cells.

Water stress is the most prominent abiotic stress that has a significant effect on crop productivity in agriculture lands around the globe. Increasing population and changing climate are likely to increase water scarcity by declining water sources, which will leads to decline in crop productivity in the world [3]. The researchers makes it very clear that reducing irrigation causes large losses in yield in maize. However, in some circumstances, this form of water deficit may allow for water savings through reduced irrigation water use at little yield cost [4]. Since these cropping and irrigation conditions are comparable to those that local farmers face, it is crucial to understand how the crops will react to a moderate water shortfall. As a result of osmotic stress, plant cells produce more solution metabolites to avoid a water shortage and a drop in turgor pressure. The metabolites, which include nitrogen components like proline and other amino acids, polyamines, and ammonium, build as a result of osmotic adjustment [5]. Organic solutions accumulate up in the cytosol and are crucial for osmotic regulation and cell retention, while a moisture deficit increases [6]. Physiological and biochemical alternation coordinated at the cellular and molecular levels results in drought resistance. Osmotic adjustment and more rigid cell walls could be part of these changes. Plant growth and productivity are negatively impacted by abiotic stress, which induces a range of morphological, physiological, biochemical, and molecular changes [7]. Drought tolerance mechanisms can be classified into three broad categories such as drought escape, drought avoidance, and biochemical tolerance of the tissues to water deficit [8]. Plants ability to withstand drought was demonstrated by the Cell Membrane Stability (CMS) experiment, which measures the integrity of cell membranes [9].

Maize has been shown to be extremely susceptible to drought [10]. The leaf area index were determined by [11] to be decreased by the degree of water stress. Likewise, deficit irrigation planning for maize is challenging without lowering production [12]. Deficit irrigation is a scheduling approach in which irrigation is purposely carried out so that the crop’s water requirements are not entirely met, and plants are allowed to take soil moisture above the water that is easily accessible in the plant roots [13].

Inoculating plants with plant growth-promoting bacteria (PGPB) has been proven to protect them from a variety of abiotic stresses [14–16]. Previous research in the literature have demonstrated that *Burkholderia spp*., *Bacillus spp*., *Pseudumonas spp*., *Azospirillum spp*., and *Rhizobium spp*. reduce the severity of drought in wheat, barley, maize, and beans [17–19]. However, the PGPB’s mode of action can be direct or indirect, and it can encourage plant growth in both stressful and non-stressful situations [20, 21]. The current experimental trial was planned to evaluate the impact of *Bacillus* spp. and deficit irrigation during early growth stage (vegetative) of maize crop.

## Materials and methods

**Fig. 1:**
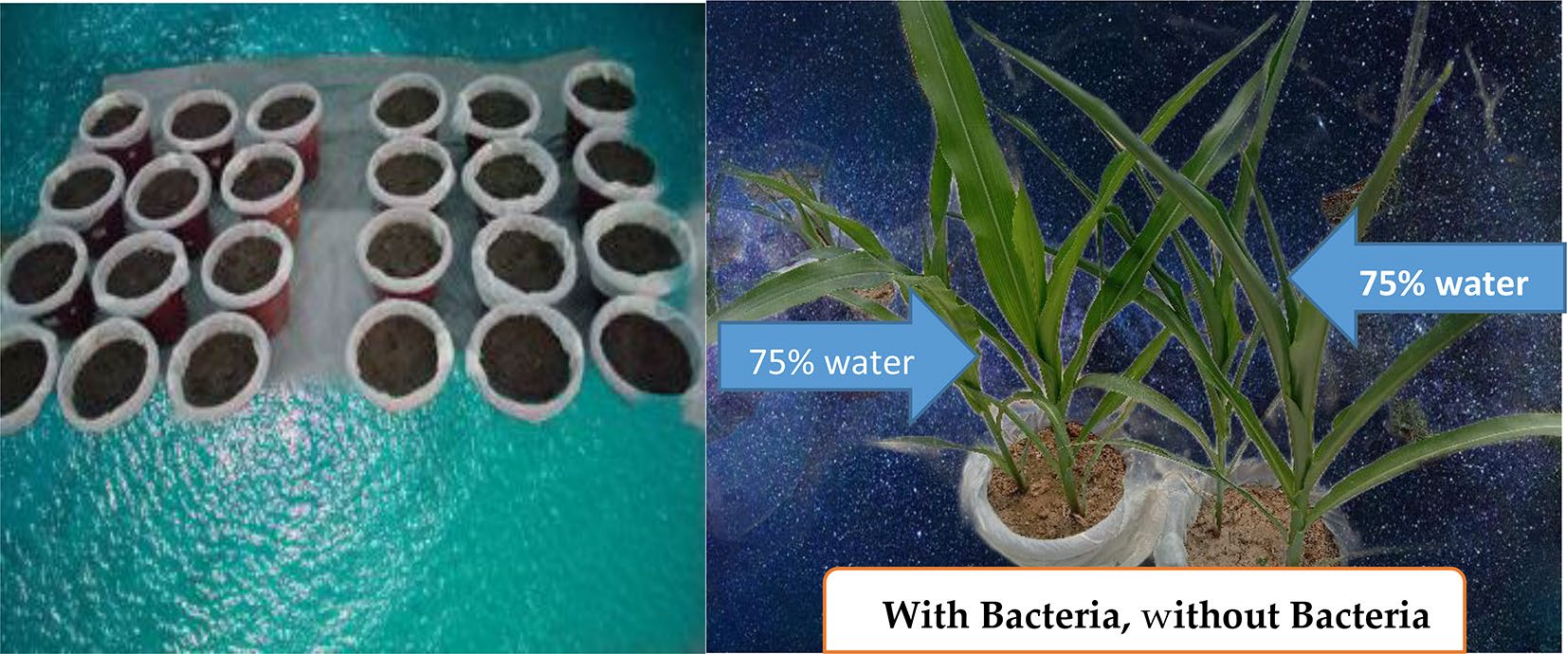
Overview of the experiment

The experiment was performed in glass house located at research site of Isparta University of Applied Sciences Isparta, Turkey in 2022. Experimental trial was conducted in Completely Randomized Design having 3 replications and a total of 24 pots were used (Fig. 1). Factorial experiment of 2 factors (Irrigation and *Bacillus*) were performed. Irrigation levels were (no stress: 100% (I_1_) and deficit irrigation: 75 (I_2_), 50 (I_3_), 25 (I_4_) % of required water amount to reach pot capacity). The product of Biotrinsic FP-30 (*Bacillus spp.* based strain) was obtained from Indigo Turkey. Based on company recommendations (36 mg (milligram) per 100 mg of seed) were applied. In this experiment the variety KWS Kerubino hybrid (*Zea mays* L., *indentata*) was used which is classified as strong grain variety in the FAO 570 mortality group and has a broad-leaved, thick and robust stem, and a body structure that is resistant to lodging. It is highly adaptable to different climatic and environmental conditions. The seed were inoculated with *Bacillus* strain prior to seeding in the pot. Total of 250 gram of seeds were inoculated with 0.072 mg of *Bacillus*. After inoculation, the seeds were mixed thoroughly for while followed by sowing.

Required amount of fertilizers (Nitrogen-Phosphorus) was calculated from MAP (Mono ammonium phosphate) and AS (Ammonium sulphate). During sowing, phosphorus was applied at a rate of 100 kg ha^− 1^ and nitrogen at a rate of 200 kg ha^− 1^ (50% at sowing and 50% at 30–40 cm height stage). Each pot of 7 kg soil was applied with 0.45 gram of MAP for phosphorus. The applied amount of nitrogen was calculated (1.3 gram N from MAP) the rest of nitrogen was calculated from AS. Each pot was supplied with 5.33 gram of AS as nitrogen base fertilizers @ 100 kg ha^− 1^. The rest of half dose nitrogen was applied at 30–40 cm height stage of crop (40 days after sowing). The examined soil properties are presented in Table 1.

**Table 1:** Soil physiochemical properties

Texture class	Clay loam	K (mg/kg)	772.2
Clay (%)	27.1	P (mg/kg)	23.5
Silt (%)	31.9	Ca (mg/kg)	8229.8
Sand (%)	41	Mg (mg/kg)	169.5
Lime (%)	28.7	Zn (ppm)	7.33
Organic Matter (%)	1.54	Cu (ppm)	2.99
pH (1:1)	7.66	Fe (ppm)	6.21
EC (µS/cm)	322	Mn (ppm)	16.2

All pots of the experiment were weighted for their initial weight and were filled with equal amount of soil. The volume of the pot was calculated as 742 cm^− 3^ (surface area as 0.28 m^− 2^). After seeding all the pots were irrigated with same amount of water and the field capacity of the pot were measured (approx. 24 h after irrigation). Afterward the remaining irrigations were performed based on water usage (evapotranspiration). All the pots of experimental trial were followed on daily basis for their weight and moisture loss and irrigation were performed according with the base amount of water (field capacity-2230 ml) by keeping the levels of irrigation under the consideration. Based on basal application of water the amount was decreased accordingly for all the treatments. Afterward, the irrigation was performed based on 30% moisture loss from the no stress treatments (I_1_) by weighing the pots.

Using [22] equation for the soil water balance, evapotranspiration (ET) from each pot was calculated (Eq. 1).

1 $$ET\hspace{0.17em}=\hspace{0.17em}I\hspace{0.17em}+\hspace{0.17em}P\hspace{0.17em}+\hspace{0.17em}{C}_{P} - \hspace{0.17em}{D}_{P} \pm {R}_{f} \pm \varDelta S$$

Where ET stands for evapotranspiration, I for irrigation water depth, P for precipitation, Cp for capillary rise, Dp for deep percolation, Rf for runoff loss, and S for change in soil water content.

Hence, there was no precipitation, capillary rise, deep percolation and runoff loss as the study was carried out in glasshouse. Downward flux below the crop root zone that was neglected because the lower side of every pot was covered with plastic bag in order to avoid drainage loss. The amount of evapotranspiration was equal to the irrigation was applied. Therefore, Eq. 1 was simplified to Eq. 2:

2 $$ET = I \pm \varDelta S$$

Equation 3 were used to compute the water usage efficiency (WUE) in the treatments [23, 24]

3 $$WUE = 100 \left(\frac{FBa}{ET}\right)$$

Where, WUE is water use efficiency, FBa is actual fresh biomass obtained and ET is evapotranspiration.

To determine the relationship between a relative decrease in evapotranspiration and a relative decrease in yield Eq. 4 was employed [25].

4 $$\left(1-\frac{FBa}{FB{\text{max}}}\right)= ky\left(1-\frac{ETa}{ET{\text{max}}}\right)$$

Where, Ky is the yield response factor, ETa and ETm are the actual and maximum evapotranspiration (mm), and FBa and FBm are the actual and maximum fresh biomass, respectively.

After 60 days the crop was harvested, fresh and dry biomass, plant height (cm), stem diameter (mm), leaf area (cm^− 2^) root length (cm), root fresh and dry weight, were measured. Scale was used to measure plant height and root length, digital calliper to measure stem diameter, and digital weight balance to measure plant weight. Equation 5 was used to calculate relative water content [26].

5 $$RWC=\frac{FW-DW}{TW-DW}\times 100$$

Where, RWC stands for relative water content. DW stands for Dry weight, FW for Fresh weight, and TW for Turgid weight.

The experiment was laid out for 2 months between 31st June-31st August. Pots were placed at the center of glass house and were tagged with proper label of corresponding treatment (Fig. 1). After 60 days of the experiment, above soil biomass (plant part) was harvested and kept in the plastics bags to avoid evaporation loss for further analysis. Root of the plant was removed with proper care from the pots by applying water in order to ease the extraction and to avoid damage. Afterward the roots were analyzed for their length fresh and dry weight.

The collected data were statistically examined using the analysis of variance method in accordance with a completely random design by using Statistix software (version 8.1). When the F-test was significant, the least significant differences (LSD) test was used to link data means [27].

## Results

### Water use efficiency

Water use efficiency (WUE) which is a measure of crop output per unit of applied water. The interactive values of both irrigation levels and bacteria were presented in Table 2 while the mean values of the individual treatments were presented in Table 4. The level of irrigation water that produced the maximum biomass (193 g pot^− 1^) and water use efficiencies (0.72 g pot^− 1^ mm^− 1^) was observed with application of I_1_ with bacteria. Keeping in consideration the least decline in biomass and WUE by the interactive effect of irrigation level I_2_ inoculated with bacteria (0.61 g pot^− 1^ mm^− 1^ WUE) when compared with 100% water level without bacteria there was no difference in water use efficiency and plant fresh biomass.

Analysis of variance shows significant effect of irrigation levels and bacteria on water use efficiency of maize crop. Plants supplied with 100% irrigation water resulted in the highest WUE (0.67 g pot^− 1^ mm^− 1^) followed by I_2_ water application with WUE (0.56 g pot^− 1^ mm^− 1^) while the lowest WUE (0.08 g pot^− 1^ mm^− 1^) was recorded in pots applied with 25% water (Table 4). According to the results obtained from this study, the interactive effect of the both factors was not significant at different irrigation levels and *Bacillus* has influenced biomass of plant and WUE.

**Table 2:** Interactive values of evapotranspiration, fresh biomass and water use efficiency (WUE), leaf area, plant dry biomass and relative water content

Treatments	ET (mm)	Fresh Biomass (g pot^− 1^)	WUE (g pot^− 1^ mm^− 1^)	Leaf area (cm^− 2^)	Plant dry biomass (g)	Relative water content (%)
I_1_B-	266.1	168	0.63	315.45b	63.16b	68.76c
I_1_B+	266.1	193	0.72	386.00a	84.00a	67.91c
I_2_B-	199.4	102	0.51	238.24d	41.83d	72.31bc
I_2_B+	199.4	123	0.61	279.44c	53.86c	74.18bc
I_3_B-	132.9	35	0.26	127.80e	18.16f	79.09ab
I_3_B+	132.9	43	0.33	136.08e	27.36e	84.81a
I_4_B-	66.4	4	0.06	47.31f	1.43 g	76.64abc
I_4_B+	66.4	7	0.10	55.30f	2.96 g	48.63d

Where, I_1_B-= 100% water + no *Bacillus*, I_2_B + = 100% water + *Bacillus*, I_2_B-= 75% water + no *Bacillus*, I_2_B + = 75% Water + *Bacillus*, I_3_B- = 50% Water + no *Bacillus*, I_3_B + = 50% water + *Bacillus*, I_4_B- = 25% water + no *Bacillus*, I_4_B + = 25% water + *Bacillus*

### Irrigation (evapotranspiration) relationships between fresh biomass

**Fig. 2:**
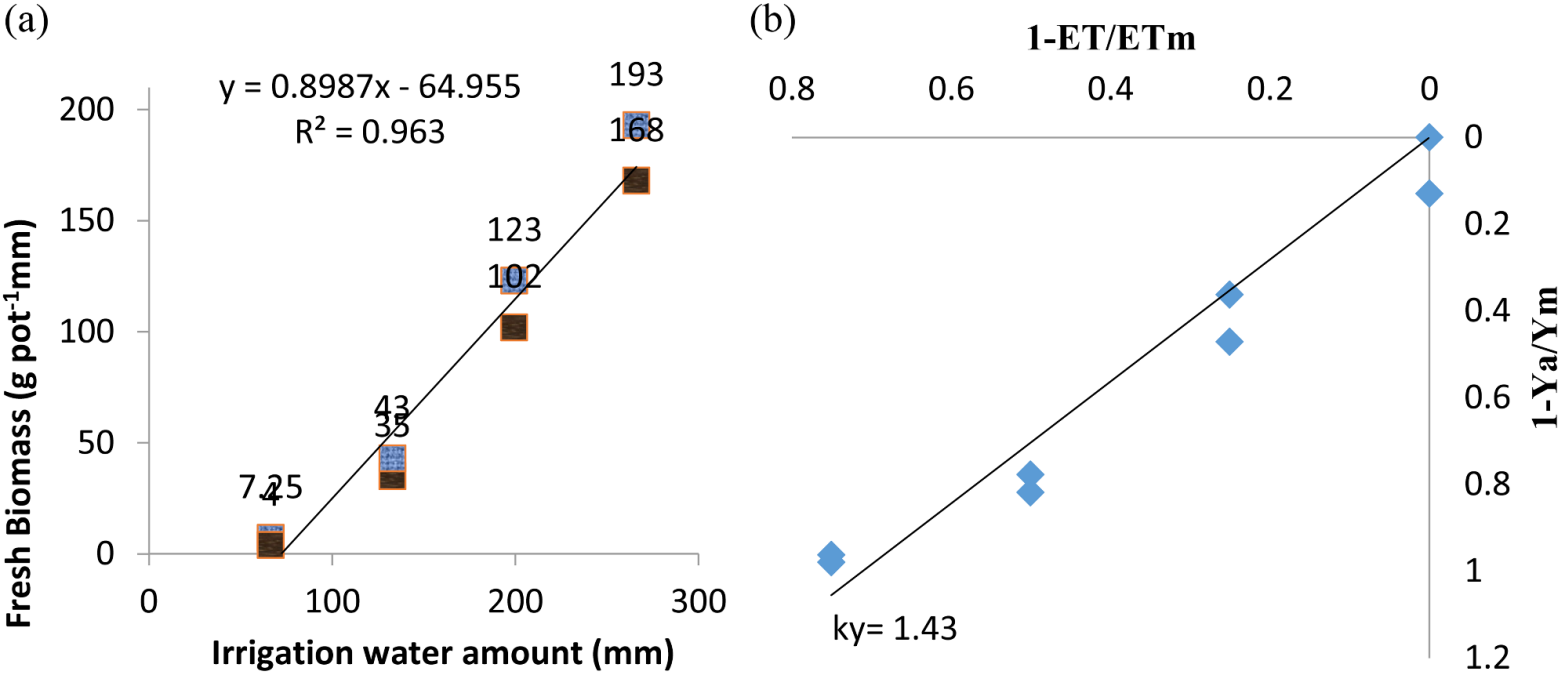
Relationships between evapotranspiration (irrigation water used), and crop biomass (**a**) and the relationships between the relative evapotranspiration deficit and the relative yield decline for maize (**b**)

Evapotranspiration and plant biomass yield have a linear relationship at 1% significant level (R^2^ = 0.963) as seen in Fig. 2. According to the findings of the study, the yield response factor (ky) was 1.43. This result illustrates that the maize fresh biomass (vegetative growth) was sensitive to water deficit.

### Plant fresh biomass (g)

Response of plant fresh biomass to various irrigation levels and biofertilizers is presented in Table 3. The effect of both irrigation and bacteria inoculation was found significant while the interactive effect of treatments was no significant. Plants applied with 100% of water (I_1_) produced more biomass (180.42 g) compared with I_2_ and I_3_ water application having fresh matter of (113 g) and (39.17 g) respectively. The growth and biomass of the plants were suppressed in case of 25% water application resulted in stunted growth and low biomass (5.63 g) production. Seeds inoculated with bacterial strain produced more vigorous biomass (91.65 g) compared with seeds without any treatment of bacteria (77.31 g).

**Table 3:** Growth attributes of maize as affected by irrigations levels and *Bacillus spp*

Treatments	Plant height (cm)	Stem diameter (mm)	Leaf area (cm^− 2^)	Plant fresh biomass (g)	Plant dry biomass (g)	Root length (cm)	Root fresh weight (g)	Root dry weight (g)
**Irrigation levels (IL)**								
I_1_	125a	18.02b	350.72a	180.42a	73.58a	92.83a	91.70a	28.66a
I_2_	104b	20.85a	258.84b	113b	47.85b	78.75b	69.83b	21.25b
I_3_	82c	13.90c	131.94c	39.17c	22.76c	74.83b	31.41c	8.66c
I_4_ LSD	44d 6.45	7.86d 1.45	51.31d 17.30	5.63d 13.48	2.20d 5.99	56.41c 55.97	6.29d 5.47	3.00d 2.40
F-value	265.0	135.34	529.64	301.19	2.39.01	6.01	438.97	212.41
**Bacteria (B)**								
B-	89.29	14.64	182.20b	77.31b	31.15	72.20b	46.10b	14.08b
B+	89.20	15.67	214.20a	91.65a	42.05	79.20a	53.52a	16.70a
LSD	ns	ns	12.23	9.53	4.24	4.25	3.86	1.69
F-value	4.56	4.53	30.76	10.17	26.99	12.58	16.51	10.73
Interaction								
IL x B	ns	ns	24.46	ns	8.48	ns	ns	ns

Where, I_1_ = 100% water, I_2_ = 75% water, I_3_ = 50% Water, I_4_ = 25% water, B- = Without Bacteria, B + = with bacteria B- = without bacteria

At the 5% level of probability, means in the same category that are followed by different letters differ significantly

### Plant dry biomass (g)

The effect of water application and *Bacillus* was found significant on plant dry biomass of maize plant (Table 3). Likewise, the interactive effect of biofertilizers and water application was also found significant. Application of 100% water showed more plant dry biomass (73.58 g) followed by 75% of water having dry matter of (47.58 g). Lowest plant dry biomass was observed in pots applied with 25% of water (2.20 g). Application of I_1_ water along with bacteria treatment resulted in pronounced effect with drier biomass followed by I_2_ water application and I_3_ water application. There were no significant differences among the means of bacterial and nonbacterial treatment (Table 3).

### Plant height (cm)

The amount of irrigation had a substantial impact on maize plant height (*P* ≥ 0.05) as shown in Table 3. However, there was no significant effect of biofertilizers on the plant height. The interactive effect of the both factors was also non-significant. By mean comparison taller plants (125 cm) was recorded under well-watered condition (I_1_) followed by I_2_, I_3_ and I_4_ irrigation treatments which was (104 cm), (82) and (44 cm) respectively.

### Stem diameter (mm)

The effect of both factors irrigation and biofertilizers was found significant for the stem diameter of plant. Plants treated with 75% (I_2_) water of field capacity resulted in maximum stem diameter (20.85 mm) followed by I_1_ irrigation (18.02 mm). Plants subjected to stress conditions (I_4_) was observed with thinner and weak stem diameter (7.86 mm). Among biofertilizers treatments, pots treated with bacteria (B+) strains shows maximum stem diameter (15.67 mm) in comparison with control (B-) having stem diameter of (14.64 mm). The comparative effect of irrigation and biofertilizers was non-significant (Table 3).

### Leaf area (cm^− 2^)

Leaf area of maize was significantly affected by both irrigation and biofertilizers. The interactive effect of the treatments was also recorded significant (Table 3). Plants treated with I_1_ treatment were observed with maximum leaf area (350.72 cm^− 2^) compared with deficit amount of water I_2_, I_3_ and I_4_ treatments with leaf area (258.84 cm^− 2^), (131.94 cm^− 2^) and (51.31 cm^− 2^), respectively. Seeds inoculated with bacterial strain also affected leaf area of maize plant. Inoculated seeds with bacteria were observed with expanded and maximum leaf area (214.20 cm^− 2^) compared with non-inoculated pots with leaf area (182.20 cm^− 2^). Among the interactions, pots inoculated with bacteria and supplied with 100% water were observed to have more leaf area (386.00 cm^− 2^) in comparison to the rest of treatments (Fig. 4).

### Root length (cm)

**Fig. 3:**
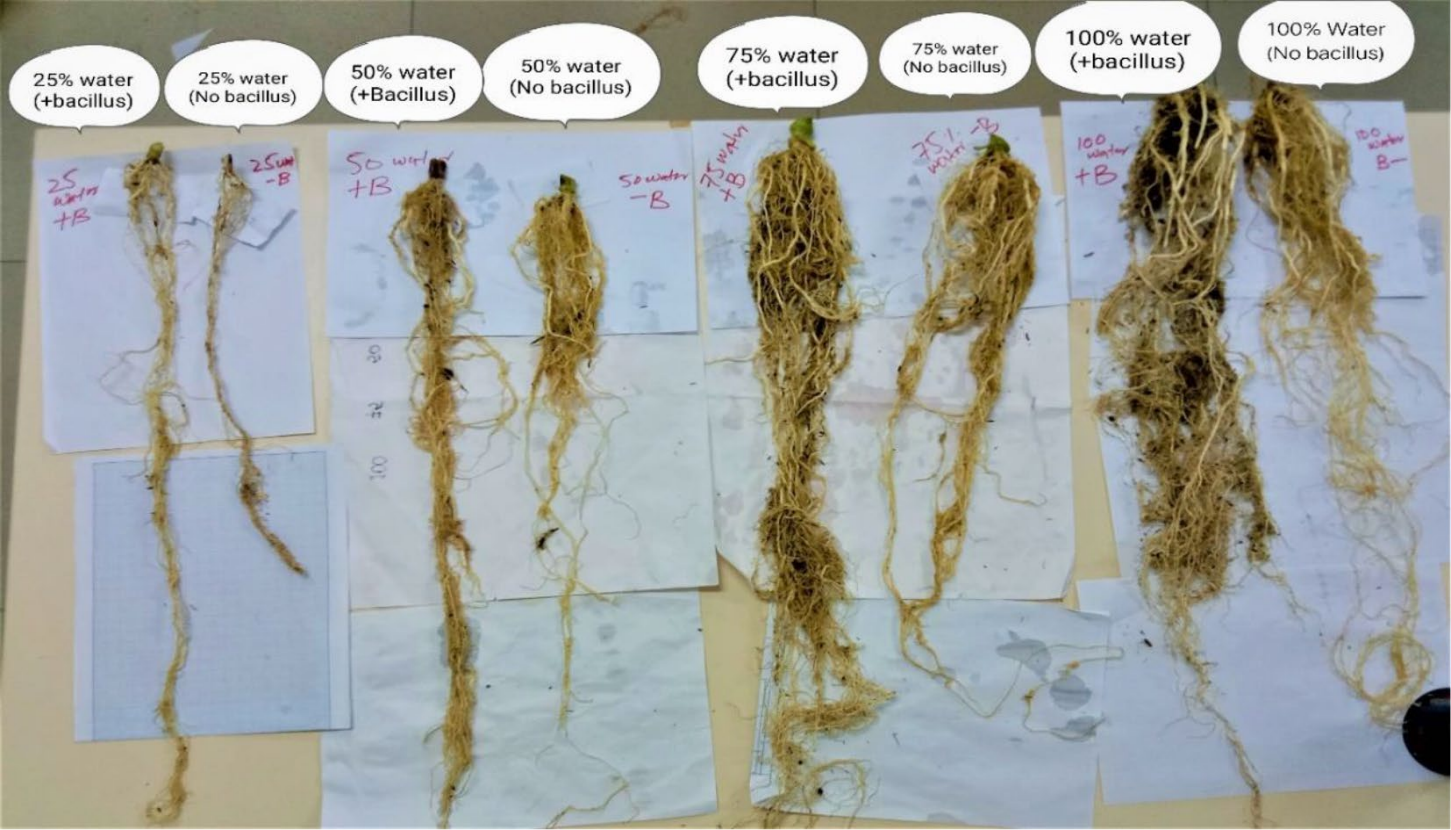
Root length of maize crop as affected by irrigation levels and bacteria

Both irrigation levels and biofertilizers has significant affected root length of maize plant. The interactive effect of irrigation and biofertilizers was found non significant (Table 3). Among irrigation levels plants treated with I_1_ water resulted in maximum root length (92.83 cm) followed by I_2_ treatment with root length of (78.75 cm) which was statistically not at far with I_3_ level of water application (74.83 cm). Plants supplied with limited water (I_4_) resulted in shorter root length (56.41 cm). Among biofertilizers, *Bacillus* inoculated plants were observed with maximum vigorous roots (79.20 cm) compared with control (72.20 cm) treatment (Fig. 3).

### Root fresh weight (g)

The effect of irrigation and biofertilizers on fresh root of weight presented in Table 3. Significant effect of both factors irrigation and biofertilizers was observed on fresh weight of root. The interactive effect of treatments was non significant. Pots applied with required amount of water (I_1_) were noted with more fresh matter (91.50 g) followed by I_2_ water application (69.83 g). Pots with least amount of water supply (I_4_) resulted in minimum fresh matter (6.29 g) production. Among biofertilizers treatments, pots with inoculated strain of bacteria produced maximum root fresh weight (53.52 g) compared with control without bacteria application (46.10 g).

### Root dry weight (g)

The effect of irrigation and bacteria inoculation was found significant on the dry weight of roots Table 3. Interactive effect of both factors was found no significant. Application of water in demand of plant (I_1_/field capacity) resulted in more dry weight of roots (28.66 g) compared with I_2_ treatment with root dry weight of (21.25 g g). Pots subjected to stress conditions (I_4_) were observed with lowest root dry weight (3.00 g). Likewise, bacteria treated seed produced maximum root dry biomass (16.70) compared with non-treated seed/pots (14.08).

### Relative water content (%)

**Fig. 4:**
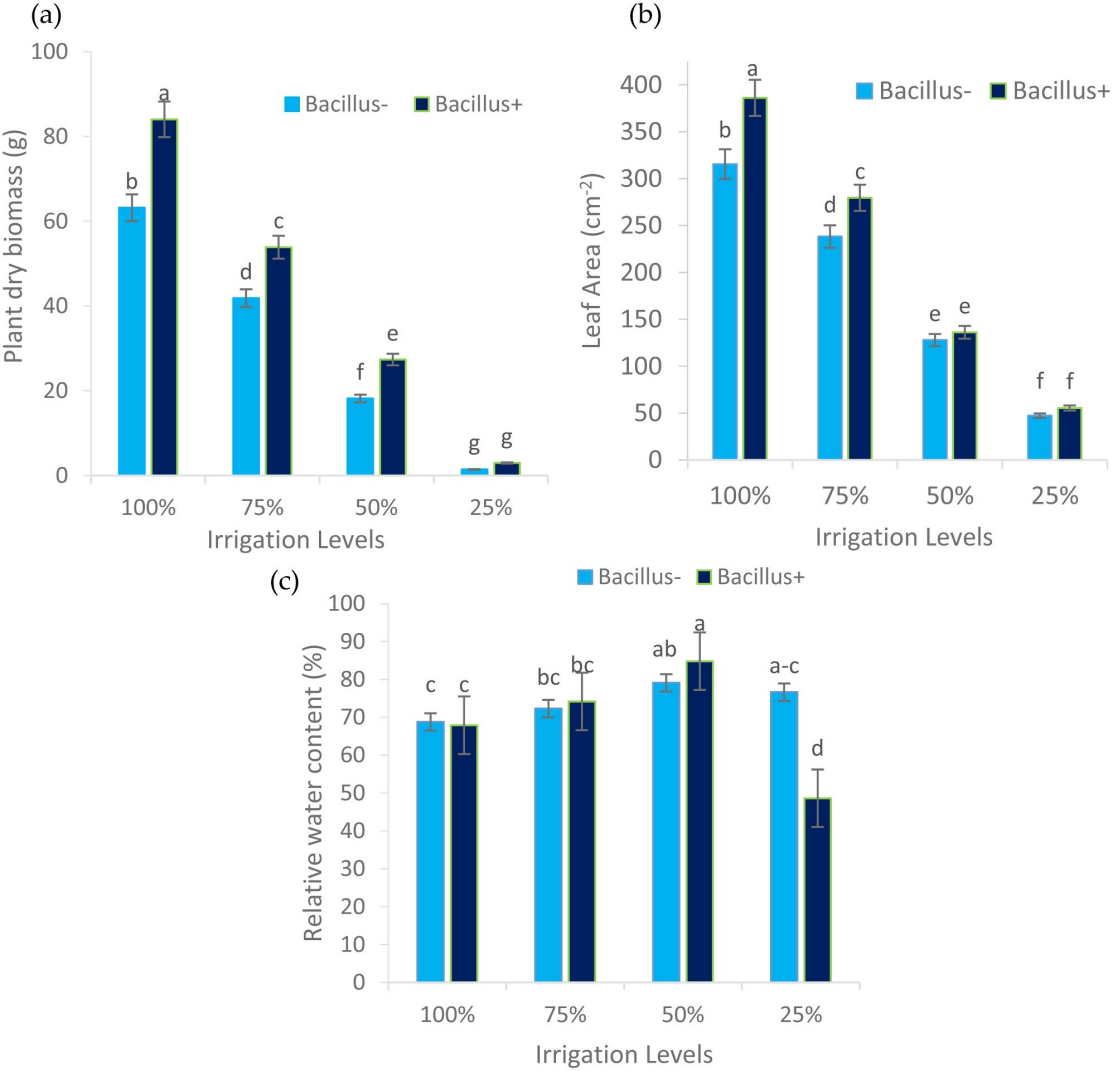
Response of plant dry biomass (**a**), leaf area (**b**) and relative water content (**c**) to irrigation levels and bacteria

Leaf relative water content as affected by irrigation levels and *Bacillus* inoculation is presented in Table 4. Irrigation and bacteria has significantly affected leaf relative water content. Integrative effect of both treatments was also significant. Plants applied with 50% (I_3_) water resulted in highest RWC (81.95%) followed by I_2_ water application. Lowest Leaf RWC was recorded in leaf of plants applied with 25% irrigation. Among bacterial treatments non inoculated plants had more RWC (74.20%) value compared with inoculated plants (68.88%). When the interactive values of both the treatments was compared highest RWC (84.81%) was recorded in plants inoculated with *Bacillus spp.* and I_3_ treatment which was statistically at far with those of non inoculated treatment and I_3_ level of irrigation (Fig. 4).

**Table 4:** Relative water content (%) and water use efficiency (g pot^− 1^ mm^− 1^) of maize as affected by irrigations levels *Bacillus spp*

Treatments	Relative water content (%)	Water use efficiency (g pot^− 1^ mm^− 1^)
**Irrigation levels**		
I_1_	68.33bc	0.67a
I_2_	73.24b	0.56b
I_3_	81.95a	0.29c
I_4_	62.63c	0.08d
LSD	7.03	0.06
F-Value	12.18	136.31
**Bacteria**		
B-	74.2a	0.36b
B+	68.88b	0.44a
LSD	4.97	0.04
F-Value	5.14	11.49
**Interaction**		
Irrigation/Bacteria	9.94	ns

Where, I_1_ = 100% water, I_2_ = 75% water, I_3_ = 50% Water, I_4_ = 25% water, B- = Without Bacteria, B + = with bacteria

At the 5% level of probability, means in the same category that are followed by different letters differ significantly

## Discussion

Plant characteristics including height, leaf area, stem diameter, and fresh and dry weight as affected by irrigation levels and *Bacillus* specie are presented in Table 3. Among the irrigations, levels the growth parameters were significantly increased with the amount of irrigation water. In the case of 100% water application highest growth in plant height stem diameter leaf area were observed followed by 75% irrigation and so on. Shortest plant height was recorded in pots applied with 25% water. This was probably of more water availability to the crop as water is an important component of plant cell and play vital role in cell division, enlargement and turgidity of the cell. These findings are in line with those of [28] who stated that highest moisture is important for cell division and growth. Stem diameter values increased parallel to increasing irrigation water [29]. The effect of bacterial inoculated seeds were found only for the leaf area and plant fresh weight of maize crop. *Bacillus spp.* treated pots resulted in more expanded leaf area compared with control (without bacteria). This could be due to due to more roots formation as a result plant extracted more water led to more leaf area and foliage production. Number of leaves on maize increased by roughly 12.4% and 8.3%, respectively, when plant growth-promoting bacteria were used as compared to the control [30]. Similar studies have been carried out and researchers determined that the growth hormones released by the bacteria were responsible for the increased cell length, cell growth, and cell division that resulted in an increase in the number of leaves. The interactive effect of the treatments were found no significant for all growth aspects except leaf area and plant dry biomass. Inoculated seeds with 100% water application resulted in highest leaf area followed by non inoculated seeds. Lowest leaf area was noted in non-inoculated plots supplied with 25% of water. The differences in the plant growth during vegetative growth may be related to the photosynthetic processes that are closely associated with leaf and whole biomass growth [31, 32].

Variability in roots characteristics of maize was measured as root length, root fresh and dry weight. Plant treated with 100% of irrigation was observed with highest root characteristics followed by 75% water application. Lowest root aspects was noted in plants treated with 25% water. The increase in root formation with water application possibly due to moist rhizosphere in pots as a result the plants had showed its potential of lengthening their roots. These outcomes are consistent with those reported by [33], who observed significant variation in roots of maize with deficit irrigation levels. Drought stress has a significant impact on root development, root properties, and behaviour [34].

In addition, plants treated with *Bacillus spp.* (inoculated plants) had the longest roots and the heaviest roots in both fresh and dry weight when compared with control plants. Numerous studies have examined PGBs, notably *Bacillus spp.*, which exhibit a variety of features that enable them to mobilize soil nutrients and synthesise phytohormones that promote plant growth [35–37]. Inoculated maize seeds with the PGPR *Azospirillum* lipoferum produced more root tips, more root branching, and longer cumulative roots [38]. By increasing the production of proline, amino acids, and soluble sugars, *Bacillus spp.* Inoculation mitigate the effects of drought, increase plant development, and improve soil water and nutrient uptake [39].

Water use efficiency as a tendency of water holding was significantly affected by irrigations levels. WUE increased as the level of irrigation increased which means, a lower level of biomass was produced per mm of water used by the plants compared with required amount of irrigations. Highest WUE was recorded in pots applied with 100% of irrigation. Water use efficiency was decreased by decreasing the level of water from 100 to 75%, 50% and 25%, respectively. Increase in WUE with increase in amount of irrigations could be due to more availability of water to the crop as a result plant has produced more biomass per unit of water applied. These results are consistent with those of [40], who reported significant increase in WUE with irrigation levels from low to high. The increase in WUE is a consequence of an overall decrease in plant transpiration caused on by a decrease in green leaf area as a result of the water shortage, which has probably reduced soil surface evaporation [41].

The combined variance analysis revealed that bacteria and irrigation practises both significantly impacted relative water content (RWC). Interactive effect of both treatments was also significant. Relative water content was decreased by increasing stress on plants. Highest RWC value was recorded in leaves of plants supplied with 50% irrigation followed by 75% and 100%. Plants exposed to stress conditions has shrinked and non turgor leaves when allow for water intake can restore their turgor therefore plants subjected to stress had highest relative water content. The findings of the current study accord with those of [42]. Among bacterial treatments, highest RWC value was recorded in leaves of plants having no inoculation in compared with inoculated plant leaves. In interactive effect of both treatments, highest RWC value was observed in inoculated plants applied with 50% irrigation which was statistical at par with non-inoculated treatment. During times of drought stress PGPB delay losses in leaf water potential, and speed up returns to control levels once water-limiting conditions are lifted [43].

## Conclusion

It can be endorsed from the outcome that, under sufficient water conditions maize crop should be irrigated with the required amount of irrigation for obtaining maximum production. In case of limited conditions, water application can be minimized to moderate level up to 75% percent by alternate approach. In our study *Bacillus spp.* showed its pronounced effect in improving growth characters of maize crop. *Bacillus spp.* inoculation increased plant biomass, root biomass, and water use efficiency of maize plant during early growth stage of maize despite of water stress and can be used under limited water condition for crop combating during moderate to lower stress conditions. In addition to this, the yield response factor (ky) value in this study was determined as 1.43 (≥ 1) which demonstrates that, maize very sensitive to a lack of water in the soil during vegetative period.

## Data Availability

The datasets used and analysed during the current study are available from the corresponding author on reasonable request.

## Abbreviations

WUE: Water use efficiency
RWC: Relative water content
I: Irrigation
B+: With bacteria
B-: Without bacteria

## References

[1] Joshi PK, Singh NP, Singh NN, Gerpacio RV, Pingali PL. Maize in India:2005 Production.

[2] Jaleel CA, Manivannan P, Wahid A, Farooq M, Somasundaram R, Paneerselvam R (2009). Drought stress in plants: a review on morphological characteristics and pigments composition. Int J Agric Biol.

[3] Abou-Basha DM, Hellal F, El Sayed S (2021). The combined effect of potassium humate and bio-fertilizers on maize productivity and quality under water stress conditions. Sci Arch.

[4] Fereres E, Soriano MA (2007). Deficit irrigation for reducing agricultural water use. J Exp Bot.

[5] Tamura T, Hare K, Yamaguchi Y, Koizumi N, Sanaa H (2003). Osmotic stress tolerance of transgenic tobacco expressing a gene encoding a membrane-located receptor- like protein from tobacco plants. Plant Physiol.

[6] Pinheiro C, Chaves MM, Ricardo CP (2001). Alterations in carbon and nitrogen metabolism induced by water deficit in the stems and leaves of *Lupinus Albus*. L J Exp Bot.

[7] Ansari WA, Atri N, Pandey M, Singh AK, Singh B, Pandey S (2019). Influence of drought stress on morphological, physiological and biochemical attributes of plants: a review. Biosci Biotechnol Res Asia.

[8] Rahman M, Ullah I, Ahsraf M, Stewart JM, Zafar Y (2002). Genotypic variation for drought tolerance in cotton. Agron Sustain Dev.

[9] Farooq S, Azam F (2002). Co-existence of salt and drought tolerance in Triticeae. Hereditas.

[10] JM, Betran J, Monneveux P, T. Drought tolerance in maize. Handb Maize: Its Biology. 2009;311–44. 10.1007/978-0-387-79418-1_16.

[11] Karam F, Breidy J, Stephan C, Rouphael J (2003). Evapotranspiration, yield and water use efficiency of drip irrigated corn in the Bekaa Valley of Lebanon. Agric Water Manage.

[12] Igbadun HE, Salim BA, Tarimo AK, Mahoo HF (2008). Effects of deficit irrigation scheduling on yields and soil water balance of irrigated maize. Irrig Sci.

[13] Payero JO, Klocke NL, Schneekloth JP, Davison DR (2006). Comparison of irrigation strategies for surface– irrigated corn in west central Nebraska. Irrig Sci.

[14] Jisha KC, Vijayakumari K, Puthur JT (2012). Seed priming for abiotic stress tolerance: an overview. Acta Physiol Plant.

[15] Gepstein S, Glick BR (2013). Strategies to ameliorate abiotic stress-induced plant senescence. Plant Mol Biol.

[16] Moreno-Galván AE, Cortés-Patiño S, Romero-Perdomo F, Uribe-Vélez D, Bashan Y, Bonilla RR (2020). Proline accumulation and glutathione reductase activity induced by drought-tolerant rhizobacteria as potential mechanisms to alleviate drought stress in Guinea grass. Appl Soil Ecol.

[17] Sandhya V, Ali SZ, Grover M, Reddy G, Venkateswarlu B (2010). Effect of plant growth promoting Pseudomonas spp. on compatible solutes, antioxidant status and plant growth of maize under drought stress. Plant Growth Regul.

[18] Farooq M, Wahid A, Kobayashi N, Fujita D, Basra SMA (2009). Plant drought stress: effects, mechanisms and management. Sustainable agriculture.

[19] Wang CJ, Yang W, Wang C, Gu C, Niu DD, Liu HX, Wang YP, Guo JH (2012). Induction of drought tolerance in cucumber plants by a consortium of three plant growth-promoting rhizobacterium strains. PLoS ONE.

[20] Ngumbi E, Kloepper J (2016). Bacterial-mediated drought tolerance: current and future prospects. Agric Ecosyst Environ Appl Soil Ecol.

[21] Kumar A, Verma JP (2018). Does plant—microbe interaction confer stress tolerance in plants: a review. Microbiol Res.

[22] James LG (1988). Principles of Farm Irrigation System Design John Wiley and sons.

[23] Howell TA, Cuenca RH, Solomon K et al. Crop Yield Response Managment of Farm Irrigation Systems. Edt. Hoffman. ASAE, Madison, Wisconsin, 1990; 312 p.

[24] Kanber R, Koksal H, Onder S, Unlu M, Sezen SM, Ozekinci B, Yazar A, Pakyurek Y (1996). Bazı Kislik Sebze Turlerinin Sulama Olanaklarinin Arastirilmasi. Cukurova Universitesi Ziraat Fakultesi Adana.

[25] Doorenbos J, Kassam AH (1986). Yield response to water. Irrig Drain Paper Rome FAO.

[26] Smart RE, Bingham GE (1976). Rapid estimates of relative water content. Plant Physiol.

[27] Steel RGD, Torrie JH. Principles and procedures of statistics 2nd ed. McGraw Hill, 1997; New York.

[28] Awwad M, El-Hedek K, Bayoumi M, Eid T (2015). Effect of potassium humate appliction and irrigation water levels on maize yield, crop water productivity and some soil properties. J Soil Sci Agricultural Eng.

[29] Ertek A, Kara B (2013). Yield and quality of sweet corn under deficit irrigation. Agric Water Manage.

[30] Soleimani FA, Naseri HR, Naseri R, Piri E (2013). Effect of plant growth promoting rhizobacteria (PGPR) on phenological traits, grain yield and yield components of three maize (*Zea mays* L.) cultivars. J Crop Ecophysiology.

[31] Song L, Jin J, He J (2019). Effects of severe water stress on maize growth processes in the field. Sustainability.

[32] Zhou H, Zhou G, He Q, Zhou L, Ji Y, Zhou M (2020). Environmental explanation of maize specific leaf area under varying water stress regimes. Environ Exp Bot.

[33] Lin Y, Watts DB, Kloepper JW, Feng Y, Torbert HA (2020). Influence of plant growth-promoting rhizobacteria on corn growth under drought stress. Commun Soil Sci Plant Anal.

[34] Franco JA. Root development under drought stress. Technology and knowledge transfer e-bulletin. 2011; Vols. 2, No. 6.Spain: Technical University of Cartagena.

[35] Niu DD, Liu HX, Jiang CH, Wang YP, Wang QY, Jin HL, Guo JH (2011). The plant growth–promoting rhizobacterium Bacillus cereus AR156 induces systemic resistance in Arabidopsis thaliana by simultaneously activating salicylate-and jasmonate/ethylene-dependent signaling pathways. Mol Plant Microbe Interact.

[36] Hardoim PR, Van-Overbeek LS, Elsas JD (2008). Properties of bacterial endophytes and their proposed role in plant growth. Trends Microbiol.

[37] Van Loon L (2007). Plant responses to plant growth-promoting rhizobacteria. Eur J Plant Pathol.

[38] El-Zemrany H, Czarnes S, Hallett PD, Alamercery S, Bally R, Monrozier LJ (2007). Early changes in root characteristics of maize (*Zea mays* L.) following seed inoculation with the PGPR *Azospirillum lipoferum* CRT1. Plant Soil.

[39] Vardharajula S, Ali SZ, Grover M, Reddy G, Bandi V (2011). Drought-tolerant plant growth promoting Bacillus spp.: effect on growth, osmolytes, and antioxidant status of maize under drought stress. J Plant Interact.

[40] Kresović B, Tapanarova A, Tomić Z, Životić L, Vujović D, Sredojević Z, Gajić B (2016). Grain yield and water use efficiency of maize as influenced by different irrigation regimes through sprinkler irrigation under temperate climate. Agric Water Manage.

[41] Karam F, Breidy J, Stephan C, Rouphael J (2003). Evapotranspiration, yield and water use efficiency of drip irrigated corn in the Bekaa Valley of Lebanon. Agric Water Manage.

[42] Naghashzadeh M (2014). Response of relative water content and cell membrane stability to mycorrhizal biofertilizer in maize. Electron J Biology.

[43] Auge RM (2001). Water relations, drought and vesicular-arbuscular mycorrhizal symbiosis. Mycorrhiza.

